# Gender-specific differences in smartphone addiction, body image, eating disorders, and physical activity: a cross-sectional investigation

**DOI:** 10.3389/fpsyg.2026.1823725

**Published:** 2026-10-02

**Authors:** Bijad S. Alqahtani, Khalid M. Alkhathami, Shajiya Sarwar Moosa, Mohammad Ahsan

**Affiliations:** 1 Department of Health Rehabilitation, College of Applied Medical Sciences at Shaqra, Shaqra University, Shaqra, Saudi Arabia; 2 Department of Anatomy, College of Medicine, Imam Abdulrahman Bin Faisal University, Dammam, Saudi Arabia; 3 Department of Physical Therapy, College of Applied Medical Sciences, Imam Abdulrahman Bin Faisal University, Dammam, Saudi Arabia

**Keywords:** body satisfaction, eating habits, females, internet addiction, males, physical exercise

## Abstract

**Introduction:**

Smartphone addiction, body image concerns, eating disorders, and physical activity are interconnected issues affecting many young people, regardless of gender. This study examines differences in smartphone addiction, body image, eating disorders, and physical activity between male and female participants.

**Methods:**

A cross-sectional study was conducted with 318 university students (168 males, 150 females) in Saudi Arabia. There average age was of 20.86 ± 3.47 years, a height of 168.24 ± 11.36 cm, and a weight of 65.26 ± 18.32 kg. Data were collected via an online survey using the Smartphone Addiction Scale-Short Version (SAS-SV), Body Image Questionnaire (BIQ-19), Eating Disorder Examination Questionnaire (EDE-Q 6.0) and International Physical Activity Questionnaire-Short Form (IPAQ-SF). All instruments demonstrated acceptable reliability (Cronbach’s α > 0.70) in previous validation studies. Statistical analysis employed Mann-Whitney U test and Kruskal-Wallis tests with a significance level of *p* < 0.05.

**Results:**

No significant differences were found in smartphones addiction (*z* = −0.64, *p* = 0.52), body image (*z* = −0.32, *p* = 0.75), physical activity (*z* = −0.61, *p* = 54). Whereas a significant difference was found in eating disorder (*z* = 2.91, *p* = 0.004), with female showing higher prevalence (88%) compare to male (76%). Sub-scales revealed significant within-group differences for all variables.

**Conclusion:**

This study revealed insignificant gender differences for smartphone addiction, body image, and physical activity levels except eating disorders. Future research should employ longitudinal designs and objective measures to clarify causal pathways and gender-specific mechanisms for all related variables.

## 1 Introduction

Smartphone addiction is increasingly recognized as a behavioral addiction characterized by excessive or compulsive use that interferes with daily life (Lu et al., 2024). Understanding gender-specific differences in smartphone addiction is crucial as males and females may differ in prevalence, patterns of use, and associated psychological factors. The prevalence of smartphone addiction varies widely across populations and regions, with meta-analytic estimates indicating rates around 30%–40% among young adults and adolescents, particularly in Asian countries (Alimoradi et al., 2022; Zhong et al., 2022). Research suggests that females may be more prone to use smartphones for social networking and emotional connection, whereas males might engage more in gaming and entertainment, which may contribute differently to addiction risk (Zheng et al., 2023). Body image refers to an individual’s perceptions, attitudes, and feelings about their physical appearance. Research consistently shows that females tend to experience higher levels of body dissatisfaction compared to males (Rounsefell et al., 2020). This is largely due to societal pressures that emphasize thinness and specific beauty ideals for women, which are perpetuated by the media and social platforms (Holland and Tiggemann, 2016). Males, while generally reporting lower body dissatisfaction, face their own pressures related to muscularity and leanness ideals (Allen and Walter, 2016). Eating disorders are complex mental health conditions characterized by abnormal eating behaviors and distorted body image. Large-scale data provide compelling evidence that smartphone addiction partially mediates the relationship between eating disorders (Ma et al., 2025). Female students generally exhibit higher risk for eating disorders and greater body dissatisfaction, while males may demonstrate higher rates of internet addiction, a closely related construct to smartphone addiction (Tayhan Kartal and Yabancı Ayhan, 2021). Physical activity plays a significant role in the context of smartphone addiction, body image, and eating disorders. Adolescents who engage in regular physical exercise demonstrated better self-control, which partially mediates their reduced dependence on smartphones (Zhang et al., 2022). Female adolescents showed a higher prevalence of low back pain associated with prolonged use of electronic devices, including smartphones (Wang et al., 2023). Similarly, university students with smartphone addiction exhibited more postural problems and muscle trigger points (Bento et al., 2020). Excessive or compulsive exercise can be a symptom or risk factor for eating disorders, particularly in sports emphasizing. Physical activity effectively reduces blood pressure in both men and women, with some evidence suggesting greater benefits in physically active males who do not use antihypertensive medication (Pescatello et al., 2019).

The available evidence suggests a complex, bidirectional association between smartphone addiction, body image concerns, eating disorders, and physical activity. As smartphone use becomes widespread, especially among younger people, understanding its psychological and behavioral effects is crucial. Smartphones, often connected with social media, can foster negative body image perceptions and contribute to the increasing rates of eating disorders. Additionally, increased screen time may reduce physical activity, further affecting overall health outcomes. Despite growing evidence associated these variables, few studies have examined their interrelationships simultaneously among Saudi university students. The Saudi cultural context, with its unique sociocultural norms regarding gender roles, body image expectations, and technology use patterns, warrants specific investigation. Understanding this gender specific pattern in Saudi Arabia is crucial developing culturally appropriate interventions. Therefore, the purpose of this study was to examine gender-specific differences in smartphone addiction, body image, eating disorders, and physical activity. The hypothesis formulated for this study were that females participants demonstrated significantly higher smartphone addiction and eating disorder compare to males, and males participants demonstrated significant higher body image satisfaction and physical activity levels compare to females participants.

## 2 Materials and methods

### 2.1 Study design

A cross-sectional study design was employed to achieve the study’s objective.

### 2.2 Study setting

This study was conducted in an online mode. The data were collected between January 2024 and May 2024 from Riyadh province Saudi Arabia.

### 2.3 Ethical approval

The studies involving humans were approved by Institutional Review Board of Shaqra University, Shaqra (HAPO-01-R-128). The studies were conducted in accordance with the local legislation and institutional requirements. The participants provided their written informed consent to participate in this study.

### 2.4 Sample size

The sample size was calculated based on a confidence level of 95%, a margin of error of 5%, a population proportion of 50%, and an estimated population size of 1,400. The following formula was used to calculate the sample size.


$$n=\frac{Z^{2}\times p\,\left(1-p\right)}{\varepsilon^{2}}$$

Where *z* = 1.96 at 95% confidence level, *p* = 0.05, and ε = 0.05 (margin of error), the calculated sample size was approximately 302 participants, and the study successfully recruited 318 participants, exceeding the required sample size.

### 2.5 Sampling technique

Non-probability sampling was used to recruit participants. An online survey link was disseminated via social networks (WhatsApp, Facebook) and email to university students. Participants were requested to circulated the survey link among classmates and other known students.

Figure 1 four horizontal bar charts present independent-samples Mann-Whitney U Test results comparing gender for SA, BI, ED, and PA measures, displaying frequency distributions, summary statistics, and mean ranks for males and females in each panel.

**FIGURE 1:**
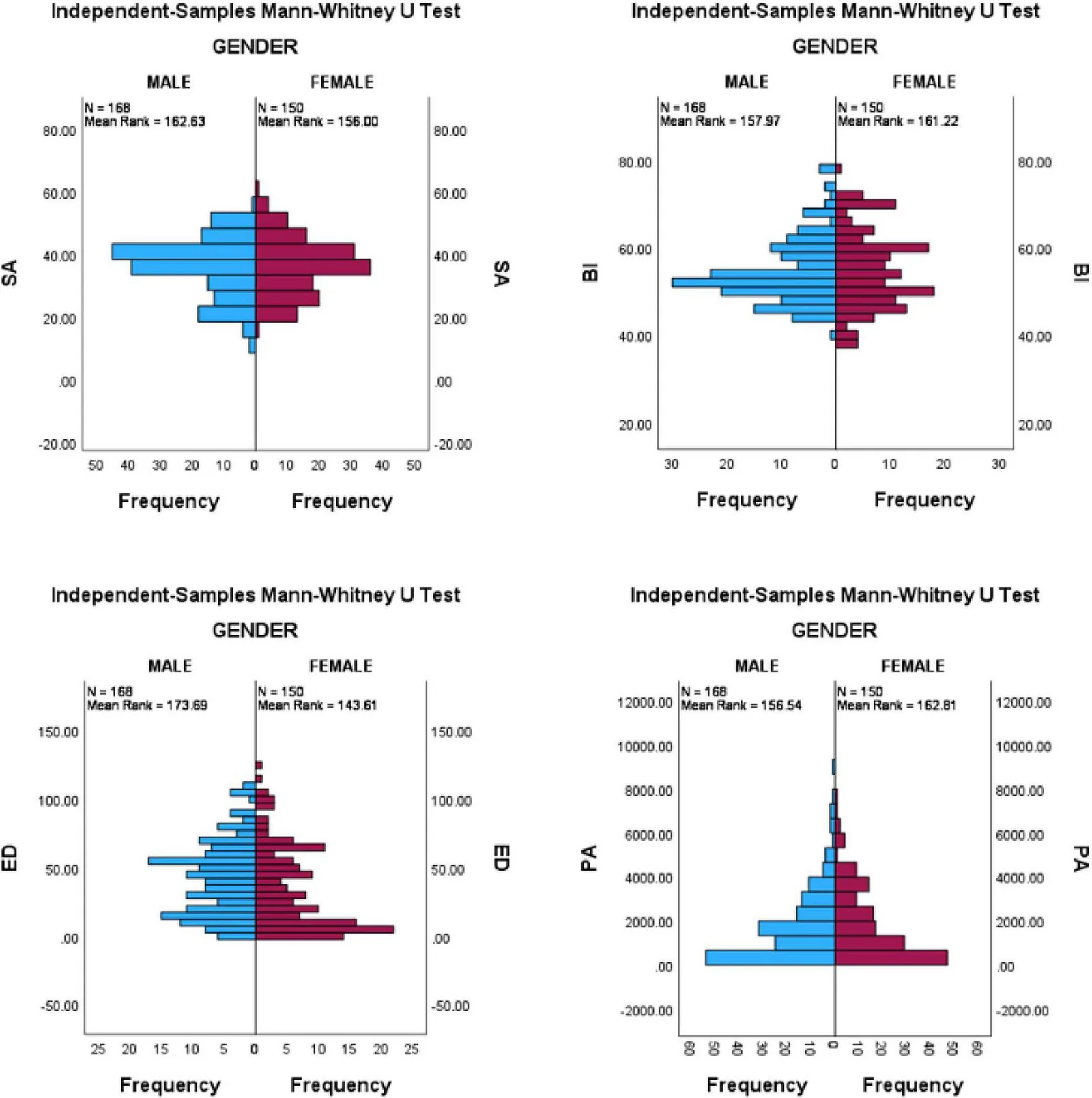
Differences between male and female participants for various parameters. SA, smartphone addiction; BI, body image; ED, eating disorder; PA, physical activity.

### 2.6 Participants

This study recruited 318 participants, with 168 male and 150 females. All participants voluntarily took part in this study.

#### 2.6.1 Inclusion criteria

University students aged 18 years or older who had used smartphone for at least 2 years.

#### 2.6.2 Exclusion criteria

Participants under 18 years old and those who had used smartphones for less than 2 years were excluded from the study. The 2-year smartphone criteria were selected to ensure participants had sufficient exposure and experience with smartphone use to develop potential addiction patterns.

### 2.7 Measures

#### 2.7.1 Smartphone addiction

The Smartphone Addiction Scale - Short Version (SAS-SV) was used to measure smartphone addiction (Kwon et al., 2013). It was designed to evaluate smartphone addiction through 10 questions focused on behavioral patterns related to smartphone use. SAS-SV was developed by Kwon et al. (2013) in and is especially useful for screening adolescents at risk of smartphone addiction, emphasizing its importance in mental health research. Males are considered addicted if they score higher than 31, while females are considered addicted if they score higher than 33 (Kwon et al., 2013). The SAS-SV has demonstrated strong psychometric properties (Cronbach’s α = 0.91 in validation studies) (Kwon et al., 2013). For the current sample, Cronbach’s α was 0.87.

#### 2.7.2 Body image

The Body Image Questionnaire (BIQ-19; Koleck et al., 2002) is a 19-item self-report instrument that assesses individuals’ perceptions and attitudes toward their body image. It assesses various aspects of body satisfaction beyond simple metrics, such as feelings, perceptions, and attitudes toward one’s physical appearance (Koleck et al., 2002). Males are considered satisfied if they score less than 56, while females are considered satisfied if they score less than 53. It is a valuable resource in psychological assessments. The BIQ-19 has been validated through extensive studies, including a principal components analysis involving over 1,000 participants, confirming its reliability and effectiveness in measuring body image across diverse populations (Koleck et al., 2002). For the current sample, Cronbach’s α was 0.82.

#### 2.7.3 Eating disorders

The Eating Disorder Examination Questionnaire (EDE-Q 6.0; Lee et al., 2011) was used to identify eating disorders within the gender population. This self-report tool is designed to evaluate eating disorder attitudes and behaviors over the past 28 days, focusing on aspects like weight control and body image. The questionnaire includes questions covering various domains, including feelings about eating, body shape, and exercise behaviors, providing insights into the individual’s relationship with food and self-image (Lee et al., 2011). For the current sample, Cronbach’s α was 0.88.

#### 2.7.4 Physical activity level

The International Physical Activity Questionnaire (IPAQ-SF; Craig et al., 2003). Short Form was used to assess physical activity levels. IPAQ-SF is a widely used questionnaire that assesses physical activity levels over the past week. It is a concise tool comprising a few questions about different types of physical activities (Craig et al., 2003). The physical activity level was determined by the estimated total physical activity in MET minutes per week. According to the WHO physical activity level classification, participants were categorized into three groups: low, moderate, and high. Test-retest reliability is ρ = 0.80. For the current sample, Cronbach’s α was 0.76.

### 2.8 Procedure

An online survey was created using Google Forms. This survey included an informed consent form and questionnaire on anthropometric data (age, weight, height, gender), marital status, duration of mobile usage, and smartphone addiction, eating disorders, body image, and physical activity level. The link to the survey was disseminated via social networks (WhatsApp, Facebook) and via email to students, and it was requested that they circulate it among their classmates and other known students. Before completing the questionnaire, they indicate their willingness to participate. They provide their anthropometric data and the best possible answers regarding mobile use, eating disorders, body image, and level of physical activity that apply to them. Approximately 20–25 min were spent attempting to complete the survey. Later, data were extracted from Google Forms and imported into MS Excel for further analysis.

To ensure data quality, we monitored completion items, checked for duplicate responses using IP address verification, and performed logic check on responses. Incomplete questionnaires (less than 90% completion) were excluded. The response rate as approximately 65% (318 completed response from approximately 490 initial participants). All data were anonymized, stored on password-protected computers, and accessible only to the research team members.

### 2.9 Statistical analysis

Statistical Analysis was conducted using the Statistical Package for the Social Sciences (SPSS) statistical Software (IBM 29.0, IBM Corp, Armonk, NY, USA) for Windows. The data were checked for outliers and normality. The Shapiro-Wilk test confirmed that the distributions of age, height, and weight were normally distributed, allowing for the use of parametric tests. However, the distributions of smartphone addiction, body image, eating disorder, and physical activity variables were not normal; thus, non-parametric tests were used. The descriptive statistical analysis was done using mean, standard deviation (SD), mean rank, sum of ranks, and graphs. Anthropometrical differences between male and female participants were determined using an independent *t*-test. A comparison between males and females for smartphone addiction, body image, eating disorders, and physical activity level was conducted using the Mann-Whitney U test. The sub-scale differences were determined by the Mann-Whitney U tests, while differences among levels of physical activity were assessed using the Kruskal-Wallis Test. Missing data were handled by listwise deletion, with less than 5% missing data overall. Effect sizes (Cohen’s d or r) were calculated where appropriate. All statistical tests were conducted at a significance level of 0.05, using a two-tailed test.

## 3 Results

The findings indicate that anthropometric measurements met the assumptions for parametric analysis, whereas the independent variables required both parametric and non-parametric testing, as indicated in Table 1. Table 2 shown significant differences were observed between males and females in height (*t* = 6.44, *p* < 0.001) and weight (*t* = 8.22, *p* < 0.001), but not in age (*t* = 0.55, *p* = 0.58). Table 3 demonstrated no significant gender differences were found in overall smartphone addiction, body image, or physical activity levels; however, eating disorder scores differed significantly (*z* = −2.91, *p* = 0.004). Table 4 revealed that significant differences were also reported across the subscales of smartphone addiction, body image, eating disorder, and physical activity for both males and females (*p* < 0.001), indicating variation in these measures across the study groups.

**TABLE 1:** Normality analysis between male and female participants for anthropometric and selected independent variables.

	Gender	Shapiro-Wilk		
Anthropometric and independent Variables		Statistic	df	Sig.
Age	Male	0.975	168	0.247
	Female	0.979	150	0.421
Height (cm)	Male	0.976	168	0.261
	Female	0.973	150	0.160
Weight (kg)	Male	0.975	168	0.246
	Female	0.974	150	0.211
Smartphone addiction	Male	0.966	168	<0.001
	Female	0.990	150	0.335
Body image	Male	0.935	168	<0.001
	Female	0.973	150	0.161
Eating disorder	Male	0.963	168	<0.001
	Female	0.905	150	<0.001
Physical activity	Male	0.860	168	<0.001
	Female	0.873	150	<0.001

This table shows that the statistical values of anthropometrical measurements are not significant. Therefore, we assume that the data are normally distributed. Thus, parametric tests were conducted. At the same time, the statistical values for different independent variables differ significantly. We assume that the data are not normally distributed, and further analysis was conducted using both non-parametric tests.

**TABLE 2:** Anthropometric differences between male and female participants.

Anthropometric variables	Gender	*N*	Mean ± SD	Std. error difference	t	*p*
Age (years)	Male	168	20.74 ± 4.09	0.346	0.553	0.580
	Female	150	20.93 ± 1.66			
Height (cm)	Male	168	171.47 ± 21.52	1.735	6.437	<0.001
	Female	150	160.30 ± 6.18			
Weight (kg)	Male	168	76.83 ± 19.11	2.216	8.220	<0.001
	Female	150	58.61 ± 20.27			

This table demonstrated that this study recruited 318 participants, with 168 male and 150 females with an average age of 20.86 ± 3.47 years, a height of 168.24 ± 11.36 cm, and a weight of 65.26 ± 18.32 kg. Significant differences between males and females in height (*t* = 6.44, *p* < 0.001) and weight (*t* = 8.22, *p* < 0.001). In contrast, no significant difference was observed between males and females in age (*t* = 0.55, *p* = 0.58).

**TABLE 3:** Differences between male and female participants for their various parameters.

Variables	Gender	Mean rank	Sum of ranks	z	Sig.	r
Smartphone addiction	Male	162.63	27,321.00	−0.642	0.521	0.04
	Female	156.00	23,400.00			
Body image	Male	157.97	26,538.50	−0.315	0.753	0.02
	Female	161.22	24,182.50			
Eating disorder	Male	173.69	29,179.50	−2.913	0.004	0.16
	Female	143.61	21,541.50			
Physical activity	Male	156.54	26,299.50	−0.607	0.544	0.03
	Female	162.81	24,421.50			

This table demonstrated that no significant differences existed between males and females in terms of Smartphone addiction (*z* = −0.64, *p* = 0.52, *r* = 0.04), body image (*z* = −0.32, *p* = 0.75, *r* = 0.02), and level of physical activity (*z* = −0.61, *p* = 0.54, *r* = 0.03) with negligible effects. In contrast, significant difference was observed between males and females for eating disorder (*z* = −2.91, *p* = 0.004, *r* = 0.16) with small effect.

**TABLE 4:** Differences between male and female participants for the sub-scales of various parameters.

			Male (*N* = 168)		z	Sig.		Female (*N* = 150)		z	Sig.
Variables	Sub-variables	*N*	Mean rank	Sum of rank			*N*	Mean rank	Sum of rank		
Smartphone addiction	Non-addicted	40	20.50	820.00	−9.546	<0.001	52	26.50	1,378.00	−10.072	<0.001
	Addicted	128	104.50	13,376.33			98	101.50	9,947.00		
Body image	Satisfied	110	55.50	6,105.00	−10.54	<0.001	68	34.50	2,346.00	−10.66	<0.001
	Unsatisfied	58	139.50	8,091.00			82	109.50	8,979.00		
Eating disorder	Present	127	71.75	9,112.00	−5.98	<0.001	132	70.86	9,354.00	−3.54	<0.001
	Absent	41	124.00	5,084.00			18	109.50	1,971		
Physical activity (MET)	Low	90	45.50		133.02	<0.001	76	38.50		123.67	<0.001
	Moderate	62	121.50				50	101.50			
	High	16	160.50				24	138.50			

This table demonstrated that significant differences existed between sub-scales of smartphone addiction (Non-Addicted vs. Addicted) {male (*z* = 9.55, *p* ≤ 0.001), Female (*z* = −10.07, *p* ≤ 0.001)}, body image (Satisfied vs. Unsatisfied) {(*z* = −10.54, *p* ≤ 0.001), female (*z* = −10.66, *p* ≤ 0.001)}, and eating disorder (Present vs. Absent) {(*z* = −5.98, *p* ≤ 0.001), female (*z* = −3.54, *p* ≤ 0.001)}, and level of physical activity (Low, Moderate, and High) {(*z* = 133.02, *p* ≤ 0.001), female (*z* = 123.67, *p* ≤ 0.001)}.

## 4 Discussion

The purpose of this study was to investigate gender-specific differences in smartphone addiction, body image, eating disorders, and physical activity. The results of this study revealed no significant differences between males and females in smartphone addiction, body image, or physical activity levels contrasting with our hypothesis. In contrast, an important difference was observed between males and females for eating disorders, with females demonstrating higher prevalence than males, supporting our hypothesis. Contrary to our hypothesis, we found no significant gender difference in smartphone addiction. This finding disagrees from some previous studies. Song et al. (2023) reported that male were slightly more likely to be affected (OR = 1.07, 95% CI = 1.03–1.12) among 36,365 Chinese medical students, Conversely, Zhong et al. (2022) found in a meta-analysis of 5,497 Asian medical students, found a higher prevalence of smartphone addiction at 41.93%, with females constituting 3,214 of the participants and showing greater addiction rates (2,181 addicted females) (Zhong et al., 2022). Menéndez-García et al. (2022) found that female children scored significantly higher on mobile phone addiction scales than males (mean 25.93 vs. 14.77, *p* = 0.03). In contrast, males scored higher on internet gaming disorder, reinforcing gender-specific addiction phenotypes (Menéndez-García et al., 2022). Our null findings may reflect the diverse disciplinary backgrounds of our participants compared to the medical students’ samples in previous studies, or potentially different use patterns in the Saudi Arabia context.

No significant gender difference was found in the body image satisfaction. This contrasts with extensive literature supporting greater body dissatisfaction among females Bodega et al. (2023); Jiménez Flores et al. (2017) and Sattler et al. (2020). Our findings may be attributed to cultural factors in Saudi Arabia, where modesty norms and less exposure to western thin-ideal media may buffer body dissatisfaction in female. Alternatively, males in our sample may experience pressures related to muscularity ideals that were not adequately captured by the BIQ-19, which focuses primarily on thin oriented body concerns. Burgon et al. (2023) meta-analyzed 21 studies (moderate quality) and found that athletes generally report lower body image concerns than non-athletes, with no significant gender differences (Burgon et al., 2023), suggesting that physical activity participation may buffer societal pressures.

The significant gender difference in eating disorders, with females showing higher prevalence, aligns with extensive literature. Capuano et al. (2025) provide a comprehensive narrative review emphasizing that females are disproportionately affected by eating disorders, with lifetime prevalence estimates ranging from 2% to 5% (Capuano et al., 2025). In contrast, males are underdiagnosed due to atypical presentations and diagnostic biases. This gender gap is supported by Yu et al. (2018), found female college students had significantly higher prevalence of disordered eating behaviors (11.6% vs. 5.7%, OR = 2.19) and food addiction (12.3% vs. 4.6%, OR = 3.04) compared to males, corroborating the epidemiological trend (Yu et al., 2018). Hansson and Schmidt (2026) critically review measurement tools for adolescent boys, highlighting that those instruments primarily validated in females may underestimate male disordered eating, particularly regarding muscularity-oriented behaviors, such as drive for muscularity, which is often neglected in traditional assessments (Hansson and Schmidt, 2026). The high prevalence in our sample (76%–88%) is concerning and may reflect the EDE-Q’s sensitivity or potential overestimation in a non-clinical population.

No significant gender difference was found in physical activity levels, consistent with some previous findings and contrasting with studies showing lower physical activity among females (Hu et al., 2021). Our findings may reflect the unique sociocultural context where Saudi females have increasing opportunities for physical activity, or the IPAQ-SF’s limitations in capturing culturally specific physical activities.

The evidence firmly establishes that smartphone addiction is a multifaceted risk factor for distorted body image, increased eating disorder behaviors, and reduced physical activity, with these effects manifesting differently across genders. Future research should employ longitudinal designs and objective measures to elucidate causal pathways and gender-specific mechanisms.

The non-significant findings for three of four parameters challenge prevailing assumptions about universal gender differences in these domains. Our results suggest that cultural context may moderate gender differences in smartphone addiction, body image, and physical activity. The significant eating disorder finding, indicates that gender remains a robust predictor in this domain, consistent with biopsychosocial models. The high eating prevalence in both genders warrants concern and suggests the need for screening and intervention programs in Saudi Arabia universities. The lack of gender differences in smartphone addiction suggests that interventions should target both genders equally.

Some limitations could impact the results of this study. Because it is a cross-sectional study, it collects data at only one point in time, which limits the ability to determine causality between smartphone addiction, body image, eating disorders, and physical activity. The study employed self-reported questionnaires or scales for these variables, which could introduce bias, as participants might tend to underestimate or overestimate their responses. It only included adults; therefore, the findings may not be fully generalizable to other groups. The lack of validated Arabic versions for all instruments may have affected measurement accuracy. Additionally, the relatively small sample size may limit the applicability of the results to the larger population of adults at Saudi universities. Participants’ existing habits, health conditions, or medications could also affect their physical activity levels and other measures, possibly confounding the results. Future studies should employ longitudinal designs with objective measure and larger, more diverse participants.

## 5 Conclusion

This study revealed significant gender differences only for eating disorders, with females demonstrating higher prevalence than males. No significant gender differences were found for smartphone addiction, body image, and physical activity. Male participants were more likely to be addicted to smartphones than females. Males were more satisfied with their body image than females. Females had higher rates of eating disorders than males. Moderate physical activity was higher in males than in females. Future research should use longitudinal designs, objective measures, and culturally adopted instruments to clarify causal pathways and gender-specific mechanisms for all related parameters.

## Data Availability

The original contributions presented in this study are included in this article/supplementary material, further inquiries can be directed to the corresponding author.

## References

[B1] Alimoradi Z. Lotfi A. Lin C. Y. Griffiths M. D. Pakpour A. H. (2022). Estimation of behavioral addiction prevalence during COVID-19 pandemic: A systematic review and meta-analysis. *Curr. Addict. Rep*. 9, 486–517. 10.1007/s40429-022-00435-6 36118286 PMC9465150

[B2] Allen M. S. Walter E. E. (2016). Personality and body image: A systematic review. *Body Image*. 19, 79–88. 10.1016/j.bodyim.2016.08.012 27636159

[B3] Bento T. P. F. Cornelio G. P. Perrucini P. O. Simeão S. F. A. P. de Conti M. H. S. de Vitta A. (2020). Low back pain in adolescents and association with sociodemographic factors, electronic devices, physical activity and mental health. *J. Pediatr*. 96, 717–724. 10.1016/j.jped.2019.07.008 31580844 PMC9432172

[B4] Bodega P. de Cos-Gandoy A. Fernández-Alvira J. M. Fernández-Jiménez R. Moreno L. A. Santos-Beneit G. (2023). Body image and dietary habits in adolescents: a systematic review. *Nutr. Rev.* 82, 104–127. 10.1093/nutrit/nuad044 37155836 PMC10711440

[B5] Burgon R. H. Beard J. Waller G. (2023). Body image concerns across different sports and sporting levels: A systematic review and meta-analysis. *Body Image*. 46, 9–31. 10.1016/j.bodyim.2023.04.007 37172557

[B6] Capuano E. I. Ruocco A. Scazzocchio B. Zanchi G. Lombardo C. Silenzi A. et al. (2025). Gender differences in eating disorders. *Front. Nutr*. 12:1583672. 10.3389/fnut.2025.1583672 40529418 PMC12171451

[B7] Craig C. L. Marshall A. L. Sjöström M. Bauman A. E. Booth M. L. Ainsworth B. E. et al. (2003). International physical activity questionnaire: 12-country reliability and validity. *Med. Sci. Sports Exerc*. 35, 1381–1395. 10.1249/01.MSS.0000078924.61453.FB 12900694

[B8] Hansson E. Schmidt M. (2026). Measuring disordered eating in adolescent boys: A systematic literature review. *Eat. Disord*. 34, 1–21. 10.1080/10640266.2024.2433325 39743500

[B9] Holland G. Tiggemann M. (2016). A systematic review of the impact of the use of social networking sites on body image and disordered eating outcomes. *Body Image*. 17, 100–110. 10.1016/j.bodyim.2016.02.008 26995158

[B10] Hu D. Zhou S. Crowley-McHattan Z. J. Liu Z. (2021). Factors that influence participation in physical activity in school-aged children and adolescents: A systematic review from the social ecological model perspective. *Int. J. Environ. Res. Public Health* 18, 3147. 10.3390/ijerph18063147 33803733 PMC8003258

[B11] Jiménez Flores P. Jiménez Cruz A. Bacardi Gascón M. (2017). [Body-image dissatisfaction in children and adolescents: A systematic review]. *Nutr. Hosp*. 34, 479–489. 10.20960/nh.455 28421808

[B12] Koleck M. Bruchon-Schweitzer M. Cousson-Gélie F. Gilliard J. Quintard B. (2002). The body-image questionnaire: An extension. *Percept. Mot. Skills* 94, 189–196. 10.2466/pms.2002.94.1.189 11883560

[B13] Kwon M. Kim D. J. Cho H. Yang S. (2013). The smartphone addiction scale: Development and validation of a short version for adolescents. *PLoS One* 8:e83558. 10.1371/journal.pone.0083558 24391787 PMC3877074

[B14] Lee P. H. Macfarlane D. J. Lam T. H. Stewart S. M. (2011). Validity of the international physical activity questionnaire short form (IPAQ-SF): A systematic review. *Int. J. Behav. Nutr. Phys. Act*. 8:115. 10.1186/1479-5868-8-115 22018588 PMC3214824

[B15] Lu X. An X. Chen S. (2024). Trends and influencing factors in problematic smartphone use prevalence (2012-2022): A systematic review and meta-analysis. *Cyberpsychol. Behav. Soc. Netw*. 27, 616–634. 10.1089/cyber.2023.0548 38979617

[B16] Ma R. Min K. Meng Z. Yang J. Zhang H. Li Q. (2025). The mediating effects of smartphone addiction in the association between mental disorders and eating disorders: Findings from a nationwide survey among Chinese youth. *BMC Public Health* 25:2401. 10.1186/s12889-025-23608-0 40624640 PMC12232801

[B17] Menéndez-García A. Jiménez-Arroyo A. Rodrigo-Yanguas M. Marin-Vila M. Sánchez-Sánchez F. Roman-Riechmann E. et al. (2022). Internet, video game and mobile phone addiction in children and adolescents diagnosed with ADHD: A case-control study. *Adicciones* 34, 208–217. 10.20882/adicciones.1469 33338245

[B18] Pescatello L. S. Buchner D. M. Jakicic J. M. Powell K. E. Kraus W. E. Bloodgood B. et al. (2019). Physical activity to prevent and treat hypertension: A systematic review. *Med. Sci. Sports Exerc*. 51, 1314–1323. 10.1249/MSS.0000000000001943 31095088

[B19] Rounsefell K. Gibson S. McLean S. Blair M. Molenaar A. Brennan L. et al. (2020). Social media, body image and food choices in healthy young adults: A mixed methods systematic review. *Nutr. Diet*. 77, 19–40. 10.1111/1747-0080.12581 31583837 PMC7384161

[B20] Sattler F. A. Eickmeyer S. Eisenkolb J. (2020). Body image disturbance in children and adolescents with anorexia nervosa and bulimia nervosa: a systematic review. *Eat Weight Disord.* 25, 857–865. 10.1007/s40519-019-00725-5 31177379

[B21] Song A. Song G. Wang H. Niu Q. Yin G. Chen H. et al. (2023). Prevalence of mobile phone addiction among medical students: A systematic review. *Am. J. Transl. Res.* 15, 2985–2998. PMC10250977 37303637

[B22] Tayhan Kartal F. Yabancı Ayhan N. (2021). Relationship between eating disorders and internet and smartphone addiction in college students. *Eat. Weight Disord*. 26, 1853–1862. 10.1007/s40519-020-01027-x 33034868

[B23] Wang J. Luo Y. Yan N. Wang Y. Shiferaw B. D. Tang J. et al. (2023). Network structure of mobile phone addiction and anxiety symptoms among rural Chinese adolescents. *BMC Psychiatry* 23:491. 10.1186/s12888-023-04971-x 37430241 PMC10332091

[B24] Yu Z. Indelicato N. A. Fuglestad P. Tan M. Bane L. Stice C. (2018). Sex differences in disordered eating and food addiction among college students. *Appetite* 129, 12–18. 10.1016/j.appet.2018.06.028 29935291

[B25] Zhang D. Su T. Lyu B. Yang Y. Zhuo X. (2022). The effects of adolescent physical activity participation on cell phone dependence: The mediating role of self-control. *Work* 72, 1289–1298. 10.3233/WOR-210702 35754248

[B26] Zheng X. Yang Y. Jiang W. He Y. Huang C. Hua Y. et al. (2023). Nonconforming gender expression and associated problematic smartphone and internet use among Chinese adolescents. *J. Behav. Addict*. 12, 817–826. 10.1556/2006.2023.00040 37639342 PMC10562814

[B27] Zhong Y. Ma H. Liang Y. F. Liao C. J. Zhang C. C. Jiang W. J. (2022). Prevalence of smartphone addiction among Asian medical students: A meta-analysis of multinational observational studies. *Int. J. Soc. Psychiatry* 68, 1171–1183. 10.1177/00207640221089535 35422151

